# Characterization of complete chloroplast genome of traditional Chinese medical plants *Paris Mairei* and its phylogenetic positions

**DOI:** 10.1080/23802359.2019.1681317

**Published:** 2019-10-30

**Authors:** Yun Song, Jin Xu, Yongjiang Zhang, MingFu Li

**Affiliations:** Institute of Plant Quarantine, Chinese Academy of Inspection and Quarantine, Beijing, China

**Keywords:** Chloroplast genome, medical plant, *Paris mairei*

## Abstract

The genus *Paris* (Liliaceae) has been used for traditional medicine in China. The wild *Paris* was on the verge of exhaustion due to illegal and immoderate exploitation coupled with environmental pollution. In order to alleviate resource pressure, *Paris mairei* can be considered as alternative sources. Here, we report the complete chloroplast genome of *Paris mairei.* The genome is 162,736 bp in length including a small single-copy region (SSC, 12,908 bp) and a large single-copy region (LSC, 84,286 bp) separated by a pair of inverted repeats (IRs; 32,771 bp). The genome contained 113 genes, including 79 protein-coding genes, 4 ribosomal RNA genes, and 30 tRNA genes. Among these genes, 16 harboured a single intron, and 2 contained a couple of introns. The overall G + C content of the cpDNA is 37.1%, while the corresponding values of the LSC, SSC, and IR regions are 35.7, 32.1, and 39.9%, respectively. The complete chloroplast genome sequence of *Paris mairei* will provide a useful resource for the conservation genetics of this species as well as for the phylogenetic studies for the genus *Paris*.

The genus *Paris* (Liliaceae) has been used for traditional medicine in China. The wild *Paris* was on the verge of exhaustion due to illegal and immoderate exploitation coupled with environmental pollution. In order to alleviate resource pressure, the overall similarity of composition-activity of different kinds of Paridis Rhizoma have been studied and *Paris mairei* can be considered as alternative sources (Wu et al. 2004). Chloroplast genome has found extensive applications in plant phylogeny (Xue et al. 2012; Dong, Xu, Cheng, Lin, et al. 2013a), genome evolution (Dong, Xu, Cheng, Zhou 2013b), high-resolution DNA barcode (Dong et al. 2012; Dong et al. 2014). Until now, no studies on the genome of *Paris mairei* have been published. Here, we reported the complete chloroplast genome (cpDNA) sequence of the *Paris mairei* (GenBank accession number: MN418229) to provide genomic information to promote its systematics research and ideas for the replacement resources of the endangered plants.

*Paris mairei* was collected from Lushui County, Yunnan province, China (98°36′41.4″E, 26°0′42.74″N) and were identified based on morphology. The voucher specimen (CAIQ160513) was deposited in the herbarium of Chinese Academy of Inspection and Quarantine, Beijing, China. Total genomic DNA was isolated from fresh leaves using the DNeasy Plant MiniKit (Qiagen, CA, USA). DNA was sheared by nebulization with compressed nitrogen gas, yielding fragments of 350 bp in length. Paired-end libraries were prepared with the Mate Pair Library Preparation Kit (Illumina, San Diego, California, USA) in accordance with the manufacturer’s instructions. Whole genome sequences were executed using Illumina Hiseq 4000 Genome Analyser. clean reads were assembled into the contigs used SPAdes 3.6.0 (Bankevich et al. 2012). Sanger sequence reads were proofread and assembled with Sequencher 4.10. All of the genes were annotated using the Dual Organellar Genome Annotator (DOGMA) software (Wyman et al. 2004). The chloroplast genome of *Paris mairei* had a length of 162,736 bp. The length of the LSC was 84,286 bp, the SSC was 12,908 bp, and the IRs were 32,771 bp. There were 113 genes in total, including 79 protein-coding genes, 30 tRNA genes, and 4 rRNA genes. 16 genes have one intron each, and 2 genes have two introns (*ycf3* and *clpP*). The G + C content was 37.1% for the whole genome, while the corresponding values of the LSC, SSC, and IR regions are 35.7, 32.1, and 39.9%, respectively.

We used RAxML8.0 (Stamatakis 2014) with 1000 bootstraps under the GTR + G model to reconstruct a maximum likelihood (ML) tree for the phylogenetic analysis of *Paris mairei* with other taxa with whole chloroplast genome sequences. The phylogenetic analysis indicated that *Paris mairei* was sister to the group including *P. luquanensis*, *P. fargesii*, *P. marmorata* (Figure 1). The complete chloroplast genome sequence of *Paris mairei* will provide a useful resource for the conservation genetics of this species as well as for the phylogenetic studies for the genus *Paris*.

**Figure 1.**
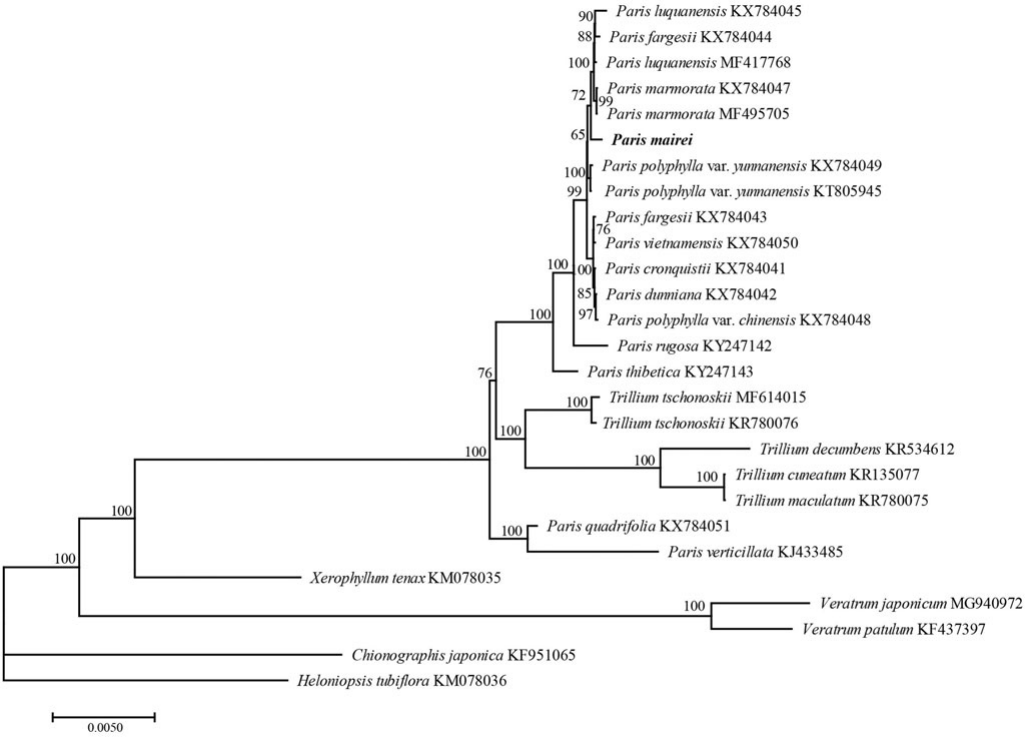
ML phylogenetic tree of *Paris mairei* and other 26 species based on the complete chloroplast genome sequences.

## Data Availability

The chloroplast genome sequence of the *Paris mairei* was submitted to Genebank of NCBI. The accession number from Genebank is MN418229.
